# Crystallinity in periodic nanostructure surface on Si substrates induced by near- and mid-infrared femtosecond laser irradiation

**DOI:** 10.1038/s41598-022-25365-1

**Published:** 2022-12-05

**Authors:** Reina Miyagawa, Daisuke Kamibayashi, Hirotaka Nakamura, Masaki Hashida, Heishun Zen, Toshihiro Somekawa, Takeshi Matsuoka, Hiroyuki Ogura, Daisuke Sagae, Yusuke Seto, Takahisa Shobu, Aki Tominaga, Osamu Eryu, Norimasa Ozaki

**Affiliations:** 1 Department of Physical Science and Engineering, Nagoya Institute of Technology, Gokiso, Showa, Nagoya, 466-8555 Japan; 2 Graduate School of Engineering, Osaka University, 2-1 Yamadaoka, Suita, 565-0871 Japan; 3 Institute of Laser Engineering, Osaka University, 2-6 Yamadaoka, Suita, 565-0871 Japan; 4 Institute for Chemical Research, Kyoto University, Gokasho, Uji, Kyoto 611-0011 Japan; 5 Research Institute of Science and Technology, Tokai University, 4-1-1 Kitakaname, Hiratsuka, Kanagawa 259-1292 Japan; 6 Institute of Advanced Energy, Kyoto University, Gokasho, Uji, Kyoto 611-0011 Japan; 7 Institute for Laser Technology, Nishi-ku, Osaka, 550-0004 Japan; 8 Institute for Open and Transdisciplinary Research Initiatives, Osaka University, Suita, Osaka 565-0871 Japan; 9 Graduate School of Science, Kobe University, Kobe, Hyogo 657-8501 Japan; 10 Department of Geosciences, Graduate School of Science, Osaka Metropolitan University, 3-3-138 Sugimoto Sumiyoshi, Osaka, 558-8585 Japan; 11 Material Science Research Center, Japan Atomic Energy Agency, Sayo, Hyogo 679-5148 Japan

**Keywords:** Applied optics, Laser material processing

## Abstract

Laser-induced periodic surface structure (LIPSS), which has a period smaller than the laser wavelength, is expected to become a potential technique for fine surface processing. We report the microscopic and macroscopic observations of the crystallinity of LIPSSs, where the characteristics such as defects generation and residual strain were analyzed, respectively. The LIPSSs were formed on a Si substrate using two different femtosecond pulses from Ti:Sapphire laser with near-infrared wavelength (0.8 μm) and free-electron laser (FEL) with mid-infrared wavelength (11.4 μm). The photon energies of the former and latter lasers used here are higher and lower than the Si bandgap energies, respectively. These LIPSSs exhibit different crystalline states, where LIPSS induced by Ti:Sapphire laser show residual strain while having a stable crystallinity; in contrast, FEL-LIPSS generates defects without residual strain. This multiple analysis (microscopic and macroscopic observations) provides such previously-unknown structural characteristics with high spatial resolution. To obtain LIPSS with suitable properties and characteristics based on each application it is paramount to identify the laser sources that can achieve such properties. Therefore, identifying the structural information of the LIPSS generated by each specific laser is of great importance.

## Introduction

Laser-induced periodic surface structure (LIPSS), which is spontaneously formed by ultra-short laser pulse irradiation, has a periodic pattern much smaller than the laser wavelength. After the first reports on the observation of laser-induced periodic structures had immerged^1^, many researches experimentally and theoretically investigated the formation mechanisms^2–11^, so far, revealing predominant physical processes governing the periodic pattern, such as surface plasmon polariton (SPP) excitation^11–13^, parametric decay^15^, and second-harmonic generation^16,17^. According to the previous works, critical laser parameters affecting the LIPSS period are wavelength, energy fluence, the number of superimposed pulses, and the ambient air pressure^18–22^. While these parameters affect the period of the LIPSS, actively controlling the period itself via coating material different from the sample substrate, has shown to be possible^23^. Recently, the use of femtosecond pulses from terahertz free-electron laser (THz-FEL)^24–28^, as well as conventional femtosecond lasers, has enabled the all-optical, contactless fabrication of structured surfaces with various periods ranging from nanoscale to microscale.

The LIPSS is a promising technique that can be applied in fine surface processing, which exceeds the wavelength limit, especially in the case using THz-FEL the periods are much smaller than one-twenties of the wavelength. The LIPSS-based processing could be non-contact, damage-less and chemical-free as advantageous characteristics compared with the conventional processing techniques such as electron beam lithography and photolithography. And LIPSS can be formed on almost all solid materials. These advantages are applicable potentially to solar cells^29–31^, hydrophilic and hydrophobic functional materials^32,33^, quantum light-emitting diodes^34^, and microscale random-laser^35^. However, the lattice states and crystallinity of the LIPSS have not yet been fully understood, although it directly influences the final device properties for such applications.

The LIPSS examined in this study were formed on the Si substrate using pulses from two different infrared femtosecond sources, a Ti:Sapphire laser and a FEL, where center wavelengths are 800 nm and 11.4 μm respectively. Utilizing Ti:Sapphire laser as a conventional light source used for LIPPS formation, and FEL as a new light source to form LIPSS, we attempt to examine the effect of different photon energies for both higher and lower than Si bandgap energies. This holds significance as the main light absorption occurs at the band-edge in general laser processing. The absorption coefficient of Si for 800 nm and 11.4 μm at 300 K is 8.5E + 02 cm^−1^^36^ and 2.32 cm^−1^^37^, respectively. Si is a typical semiconductor material, which is applied for various opt- and power- devices such as solar cells or transistors, where the performance of these devices is controlled by the electronic states. Therefore, it has been chosen as the substrate for the samples used in this study. We investigated the crystallinity of the LIPSS by Synchrotron high-energy x-ray diffraction (XRD), through a correlation with crystallographic analysis of transmission electron microscope (TEM) observation^38–41^.

## Methods

### LIPSS formation

We used Ti:Sapphire laser (wavelength λ: 800 nm, pulse duration: 100 fs, repetition frequency: 1 kHz) and MIR-FEL^42,43^ (λ: 11.4 μm, micro-pulse duration: 500 fs, repetition frequency of micro-pulses: 2856 MHz, macro-pulse duration: 2 μs, repetition frequency of macro-pulses: 2 Hz) for the formation of LIPSS. Si (100) substrates were used as irradiated materials. The energy fluences per pulse were 0.092 J/cm^2^ in the case of irradiation using Ti:Sapphire laser, and 9.9 J/cm^2^ (macro-pulse) and 0.0017 J/cm^2^ (micro-pulse) in the case of MIR-FEL, respectively. The number of the laser pulses irradiated at the same point was calculated to be 30 for the Ti:Sapphire laser and 14 for the MIR-FEL.

### Crystallinity characterization

The LIPSSs were analyzed by TEM (*JEM-2100EX*, JEOL) and high-energy XRD using two-dimensional detector^44,45^. The samples for TEM observation were prepared using a focused-ion-beam (FIB) processing machine (*JEM-9320FIB*, JEOL). The FIB process was applied to thin the sample and make observation of the transmitted images possible. During the FIB process, C was deposited to protect the sample surface and Ga was deposited by the processing ions to thin the sample. The thickness of the mixed layer can be calculated to be approximately 50 nm. The FIB process was performed before the TEM observation, and not performed on the sample for the XRD measurement.

**Figure 1:**
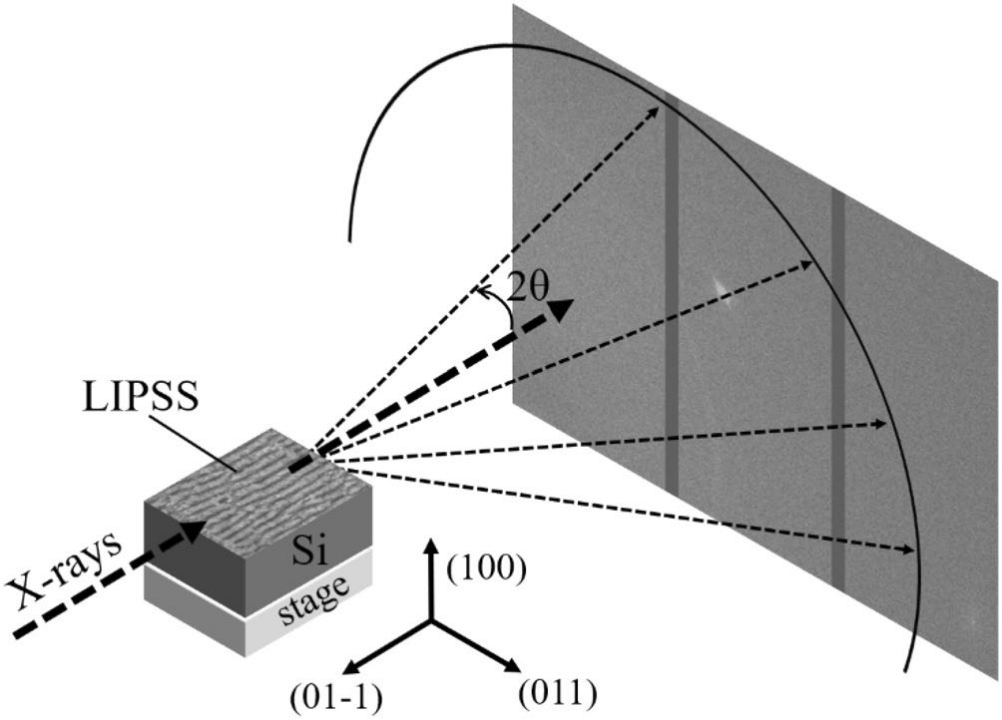
Schematic diagram for the XRD measurement setup. We performed XRD analysis using high-energy x-rays (30 keV). Transmitted x-ray diffraction light was detected by two-dimensional detector. The dotted arrows show the direction of the x-rays and the solid arrows represent the crystal orientation of Si substrate. The measurement position was adjusted by moving a stage under the Si substrate.

The XRD analysis using two-dimensional detector provides not only the information of lattice constant but also the distribution of crystal azimuth. The XRD measurement was performed using beamline BL22XU in SPring-8. The photon energy of the x-rays was 30 keV, and the beam size was 20 μm. Transmitted x-ray diffraction spot/ring was detected by 2D detector (PILATUS300K, DECTRIS) as shown in Fig. 1, where the dotted arrows show the direction of the x-rays and the solid arrows represent the crystal orientation of Si substrate, respectively. The angular resolution of this XRD system is high at a 2*θ* level of 0.02°–0.04°.

## Results

### Surface analysis by SEM observation

**Figure 2:**
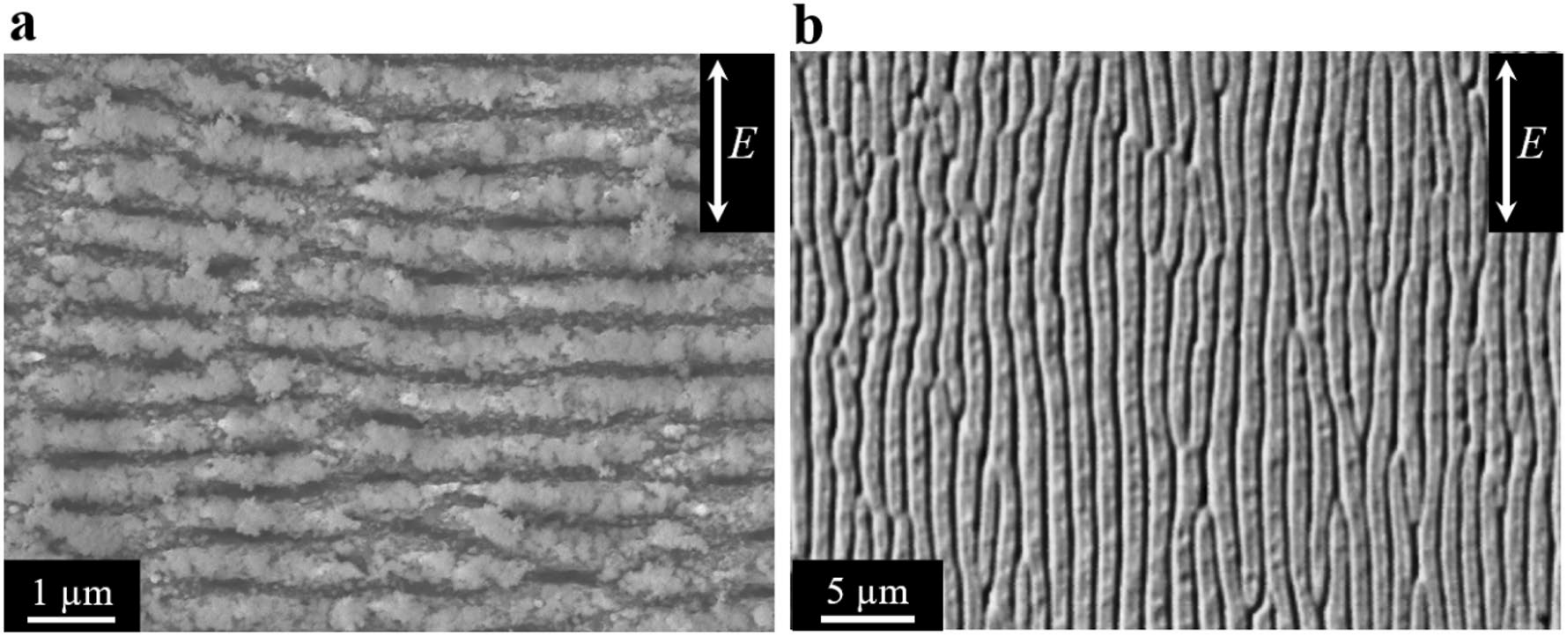
Surface SEM images of LIPSS formed using (**a**) Ti:Sapphire laser and (**b**) MIR-FEL. LIPSSs were formed perpendicular in the case of Ti:Sapphire laser and parallel in the case of MIR-FEL to the direction of the laser polarization, which is represented as *E* in the figure. The periods of the LIPSS were approximately 582.5 ± 35 nm and 1.09 ± 0.35 μm, respectively.

The surface images of LIPSS taken by a scanning electron microscope (SEM) are shown in Fig. 2. Figure 2a,b show the LIPSSs formed by Ti: sapphire laser and MIR-FEL, respectively. The applied laser was linearly-polarized and the direction of the laser polarization is shown in the figure as *E*. In the case of Ti:Sapphire laser, the LIPSS was formed perpendicular to the *E* and the period of the LIPSS was 582.5 ± 35 nm, which was measured by Fourier-transformed image. The period of the LIPSS corresponds to 70% of the laser wavelength. Meanwhile, the LIPSS formed by MIR-FEL are parallel to the *E* and the period is 1.09 ± 0.35 μm, which corresponds to a tenth of the applied laser wavelength. In some previously published literatures, it has been reported that the direction of the LIPSS depends on the irradiated materials or the ratio of LIPSS-period to the applied laser wavelength^46–48^. Bonse et al*.* reported that the LIPSS with a bit shorter period than the applied laser wavelength (0.5λ < period < λ) was formed parallel to the *E*, and the LIPSS with shorter period than half the laser wavelength (< 0.5λ) was formed perpendicular to the *E* in the case of Si substrate. Our results match reasonably well with these previous reports^46,47^.

### Micro-characterization by TEM observation

**Figure 3:**
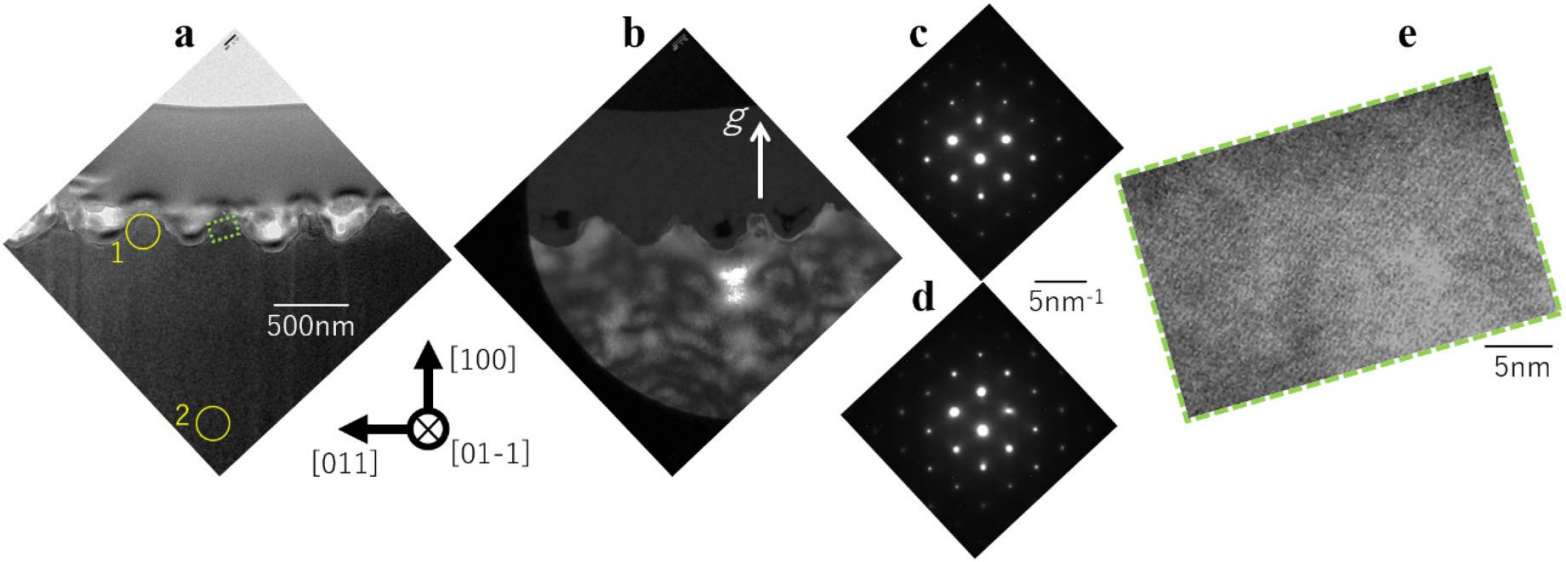
TEM images of the LIPSS induced by Ti:Sapphire laser. (**a**) Bright-field TEM image, (**b**) dark-field image, (**c**) diffraction pattern of area 1, (**d)** diffraction pattern of area 2, (**e**) high-resolution image of the square-marled area in figure (**a**).

Cross-sectional TEM images of LIPSS induced by Ti:Sapphire laser are presented in Fig. 3, where Fig. 3a is a bright-field image, Fig. 3b is a dark-field image (*g* ║ [100]_Si_), Fig. 3c,d are selected area electron diffraction (SAED) patterns detected at the area 1 and 2 shown in Fig. 3a,e is high-resolution TEM image at the square-marked area in Fig. 3a. The surface layer of approximately 50 nm is C and Ga deposited during the FIB process. No defects are detected and the spot patterns were detected from both LIPSS and substrate areas. These results demonstrate that the LIPSS induced by Ti:Sapphire laser kept crystalline, comparable to the substrate. Moreover, the high-resolution TEM images presented in Fig. 3e depict that the Si atoms arrangement is kept regularly.

**Figure 4:**
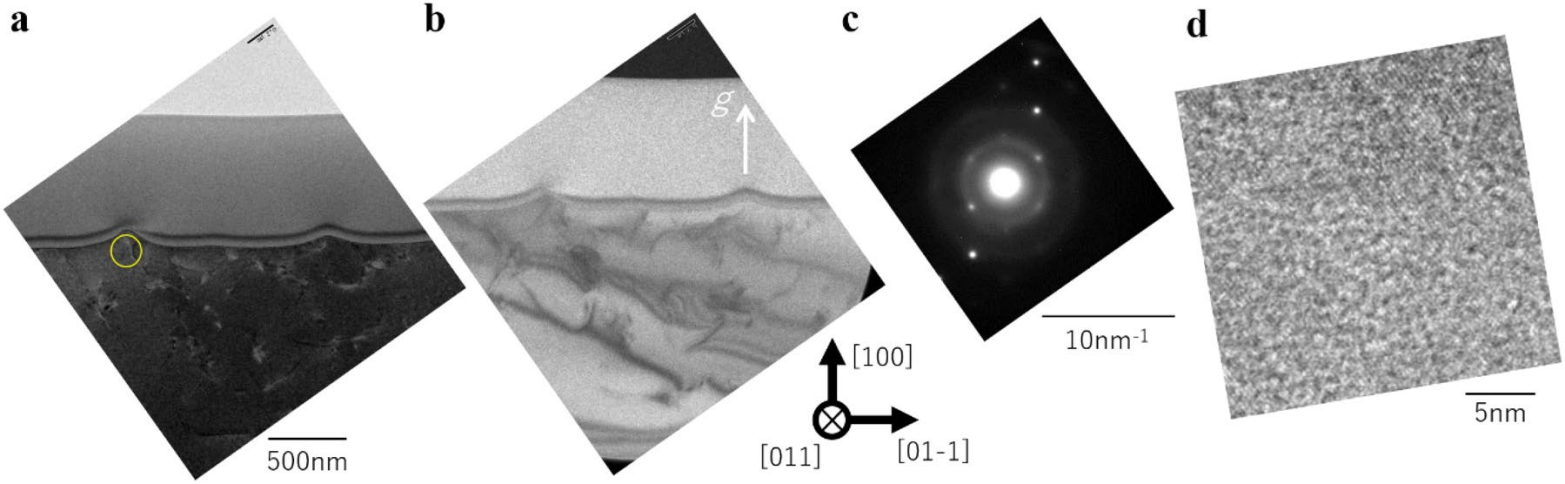
TEM images of the LIPSS induced by MIR-FEL. (**a**) Bright-field TEM image, (**b**) dark-field image, (**c**) diffraction pattern and (**d**) high-resolution image of the marled area in figure (**a**).

Figure 4 shows the TEM images of LIPSS induced by MIR-FEL, where Fig. 4a is a bright-field image, Fig. 4b is a dark-field image (*g* ║ [100]_Si_), and Fig. 4c is SAED pattern detected at the circle area shown in Fig. 4a,d is high-resolution TEM image at the marked area in Fig. 4a. Figure 4a, b depict generation of the dislocation-like defects at LIPSS area and until the depth approximately 1 μm. Similar to previous reports on the defect evaluation of Si^49^, we believe that these defects are clusters of point defects, such as dislocation loops or voids. These defects can soccurr along the slip plane (111) when local temperature rise was happened by laser irradiation. The SAED pattern with both spot and ring depicts that the LIPSS kept crystalline but had local random orientation. These patterns have been reported to be observed at the amorphous area or at randomly oriented nanoparticles^50^. Our results of the high-resolution TEM observation shown in Fig. 4d also match these reports. Figure 4d shows the regular atomic arrangement, though including some local round-shaped disarrangement in 2–3 nm size. The results depict that the LIPSS was crystalline at the level that the atomic arrangement can be observed, but also some local nanoparticles rotated the alignment slightly.

### Macro-characterization by high-energy XRD analysis

**Figure 5:**
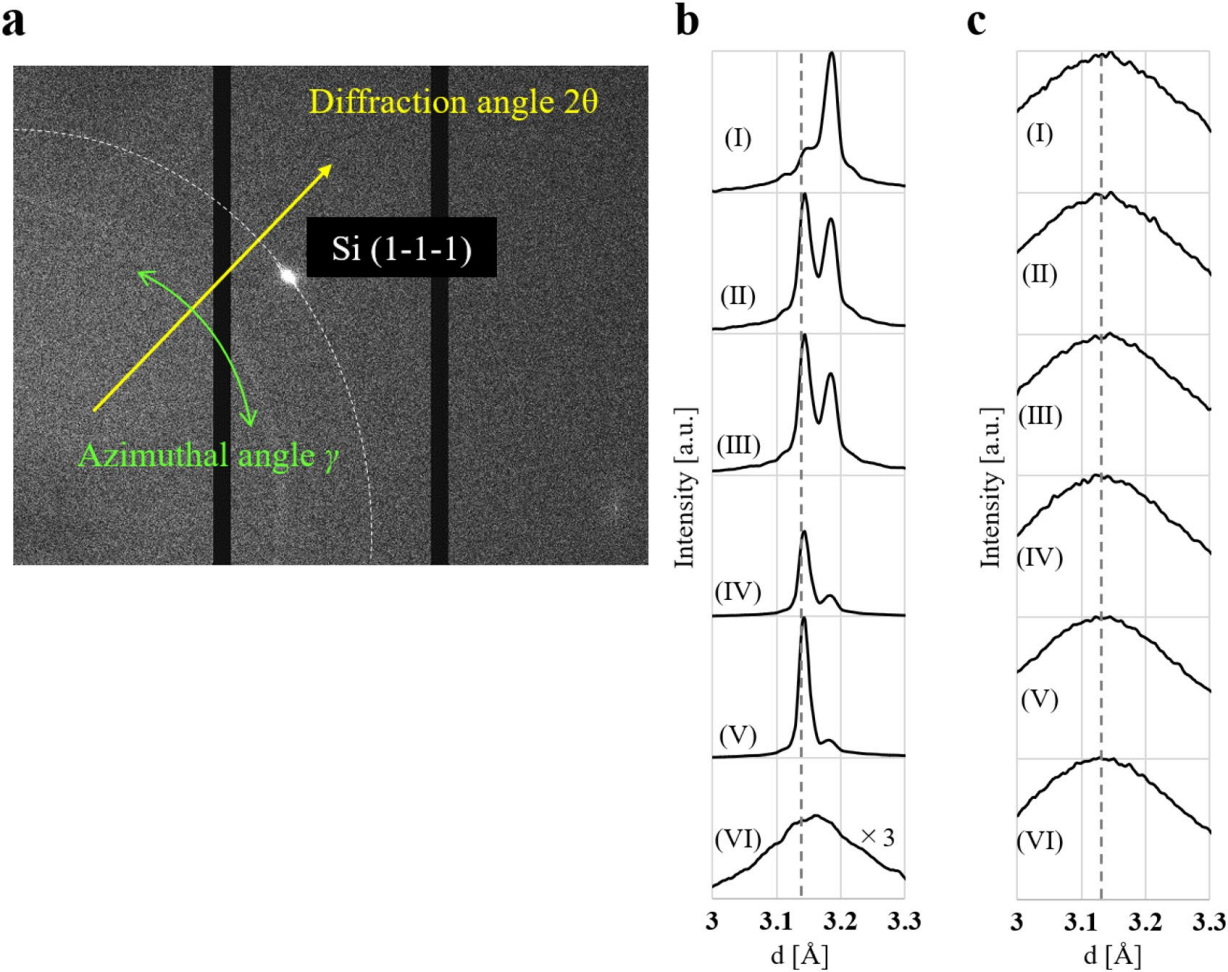
Two-dimensional XRD pattern and the lattice spacing. (**a**) Two-dimensional XRD pattern of the LIPSS formed by Ti:Sapphire laser. (**b**,**c**) The lattice spacing-intensity profiles of the LIPSS formed by Ti: sapphire laser and MIR-FEL. The lattice spacing-intensity profiles show the depth dependence. The center position of the x-ray beam was at (I) 20 μm above from the top surface of the LIPSS, (II) 10 μm above from the top surface of the LIPSS, (III) the top surface of the LIPSS, (IV) 10 μm below the top surface of the LIPSS, (V) 20 μm below, (VI) 200 μm below the top surface of the LIPSS.

Two-dimensional (2D) XRD pattern of LIPSS induced by Ti:Sapphire laser is shown in Fig. 5a. The arrows indicate the diffraction angle (2*θ*) and the crystal azimuthal angle (*γ*), respectively. The diffraction peak of Si (1–1–1) plane was detected in the measurement condition shown in Fig. 1. The spot-shaped peak depicts that the LIPSS is crystalline oriented, however, the extension along like a part of the halo-pattern shows the crystalline fluctuation slightly. The lattice spacing (*d*) was calculated using Bragg’s equation, where the wavelength of incident x-rays of 0.4135 Å. For the LIPSS induced by the Ti:Sapphire laser pulses, the detected *d* value is 3.16049 Å whereas, in the case of the MIR-FEL, it is 3.13852 Å. The *d* value of the substrate, which is detected at 200 μm below the top surface of the LIPSS, was calibrated to 3.135 Å as Si (1–1–1) lattice spacing^51^. The lattice spacing of the LIPSS induced by Ti:Sapphire laser increased by 0.02549 Å compared with the substrate, whereas the difference between the LIPSS induced by MIR-FEL and substrate was just 0.00352 Å. Figure 5b,c show the lattice spacing-intensity profile of the LIPSS induced by Ti:Sapphire laser and MIR-FEL, and (I)–(VI) depict the depth dependence, in which the center position of the x-ray beam was at (I) 20 μm above the top surface of the LIPSS, (II) 10 μm above the top surface of the LIPSS, (III) at the top surface of the LIPSS, (IV) 10 μm below the top surface of the LIPSS, (V) 20 μm below the top surface of the LIPSS, and (VI) 200 μm below the top surface of the LIPSS. The dotted line indicates 3.135 Å. All the peaks detected from MIR-FEL and those detected 200 μm below the top surface of the Ti:Sapphire-LIPSS were broad because the samples were set slightly-shifted from the position that satisfies the Bragg’s equation, where this is to detect small change. As a result, at the position 200 μm below the top surface of LIPSS the diffraction is caused by the substrate area, not-including the LIPSS area. The profile of LIPSS induced by Ti:Sapphire laser demonstrates two sharp peaks at 3.160 Å and 3.135 Å. The lattice spacing at 20 μm above the top surface of the LIPSS, which means that it was diffracted only by the LIPSS area, was 3.16049 Å as mentioned above. With moving the diffraction area from LIPSS to the substrate, the intensity of the peak at 3.160 Å decreased while the peak at 3.135 Å became dominant. These results depict that residual tensile strain was obtained at the area of the LIPSS induced by Ti:Sapphire laser, and the strain value was calculated as 0.008^52^. According to theoretical research on the multi-physical model of LIPSS formation^53^, nanocavities are formed especially at the tips of LIPSS. Hence, the nanocavities might cause the lattice expansion and the residual tensile strain. On the other hand, only one peak was detected from the LIPSS induced by MIR-FEL, while its peak slightly shifted from 3.135 to 3.138 Å moving the center position of the x-ray beam from LIPSS to the substrate.

## Discussion

The results of LIPSS formed by Ti:Sapphire laser, TEM observation demonstrated a crystallinity comparable to the substrate without significant defects, and the XRD measurement depict that residual tensile strain was generated in (1–1–1) plane at LIPSS area. The high-energy XRD measurement provided unknown information about residual strain in LIPSS with high spatial resolution at a level less than 0.1 Å, where the measurement resolution of the equipment setup is 0.004 Å. The angular resolution of this XRD observation is high enough at 2*θ* levels of 0.02°–0.04°, corresponding to atomic displacements of 5 × 10^–3^ Ȧ or less. This residual strain would not affect electronic or optical device properties significantly, however, the pivotal point is that it is possible to evaluate the strain with this high resolution.

For the case of LIPSS formed by MIR-FEL, it exhibited defects while some local nanoparticles rotated the alignment slightly. For amorphous-like state with rotated nanoparticles, the distance to first neighbor atom is the same as in crystalline Si. Therefore, the crystal state evaluated by TEM shows a good correlation with the result of XRD which is that the lattice spacing of (1–1–1) plane did not present the difference between LIPSS and substrate. Moreover, the generation of defects might reduce the residual strain.

The crystallinity difference could originate from the difference in the optical absorption process during LIPSS formation. In the case of Ti:Sapphire laser, which the photon energy is higher than Si bandgap, the absorption occurred through the electron transitions from the valence band to the conduction band. On the other hand, in the case of MIR-FEL, whose photon energy is much lower than the bandgap. These lasers differ significantly in wavelength and pulse structure. Therefore, their LIPSSs could be caused by each different predominant absorption process, which is not yet fully understood. One possible process is, for example, the nonlinear absorption enhancement discovered recently in highly repetitive MIR-FEL pulses (2856 MHz) irradiation^54^.

These defects or residual strain in LIPSS, in general, are not supposed to have a significant impact on the optical or electronic device properties where they are utilized such as solar cells or LED, the residual strain and consequent stress may affect the mechanical properties of the device surface. Continuous investigation of any positive and negative effects on the device properties due to these defects in detail is important and we will intend to as our future research.

## Conclusion

We demonstrated that the combination of analysis by TEM and high-energy XRD complemented each other as the micro-scopic and macro-scopic analysis, respectively. These evaluations, where micro characteristics such as defects or atomic arrangement by TEM and macro characteristics such as residual strain by XRD, provide valuable information in order to utilize LIPSS for prospective applications in devices. We also demonstrated the crystallinity difference of LIPSS depending on the applied laser. We used Ti:Sapphire laser, whose photon energy is higher than the bandgap energy of Si, and MIR-FEL, whose photon energy is lower than the bandgap energy of Si. The LIPSS induced by Ti:Sapphire laser kept high crystalline but occurred slight residual strain. In contrast, the LIPSS induced by MIR-FEL generated the defects but no observable strain. The crystallinity difference could be caused by each different predominant absorption process during LIPSS formation, which is not yet fully understood. And the period of the LIPSS strongly depends on the applied laser, since the laser wavelength is known as the most effective parameter. We can prioritize the characteristic of the LIPSS, such as periods, strain and effects, for the expecting application and the desired structures (Supplementary Information S1).

## Supplementary Information

Supplementary Information.

## Data Availability

The datasets used and/or analyzed during the current study available from the corresponding author on reasonable request.
