# Supplementary material for: Crystallinity in periodic nanostructure surface on Si substrates induced by near- and mid-infrared femtosecond laser irradiation
Source: Sci Rep. 2022 Dec 5;12:20955. doi: 10.1038/s41598-022-25365-1 (PMC9722692; doi:10.1038/s41598-022-25365-1)
Supplement: Supplementary file 1 — Supplementary Information. [file 41598_2022_25365_MOESM1_ESM.pdf]

Original figures of Fig. 2 (Surface images)

a

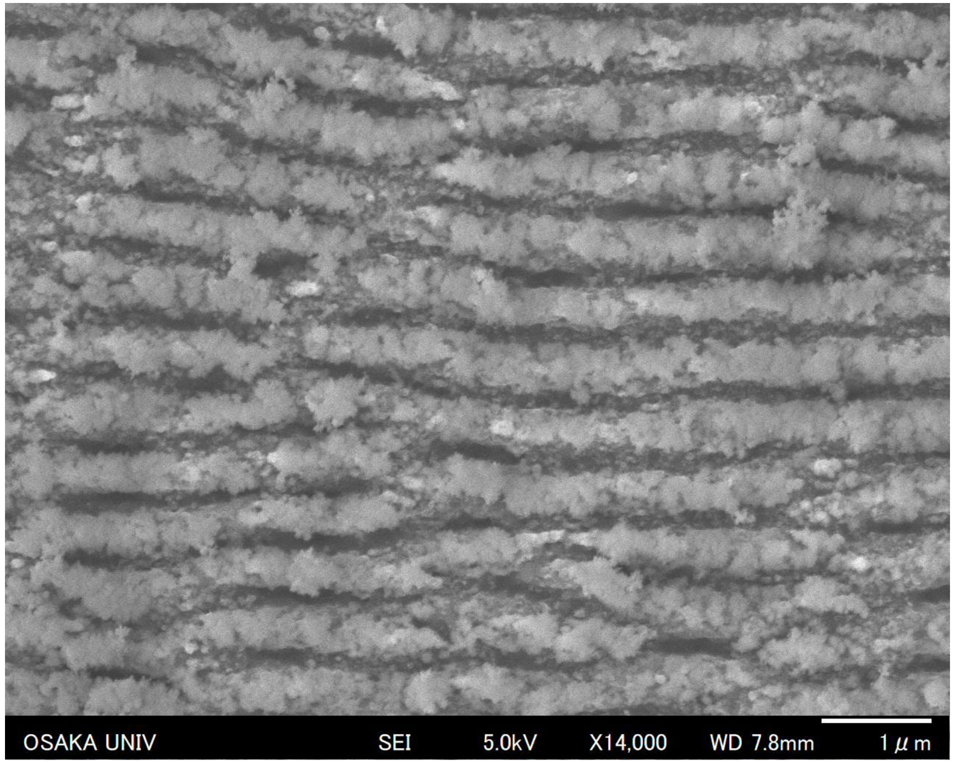

Ti: sapphire laser

b

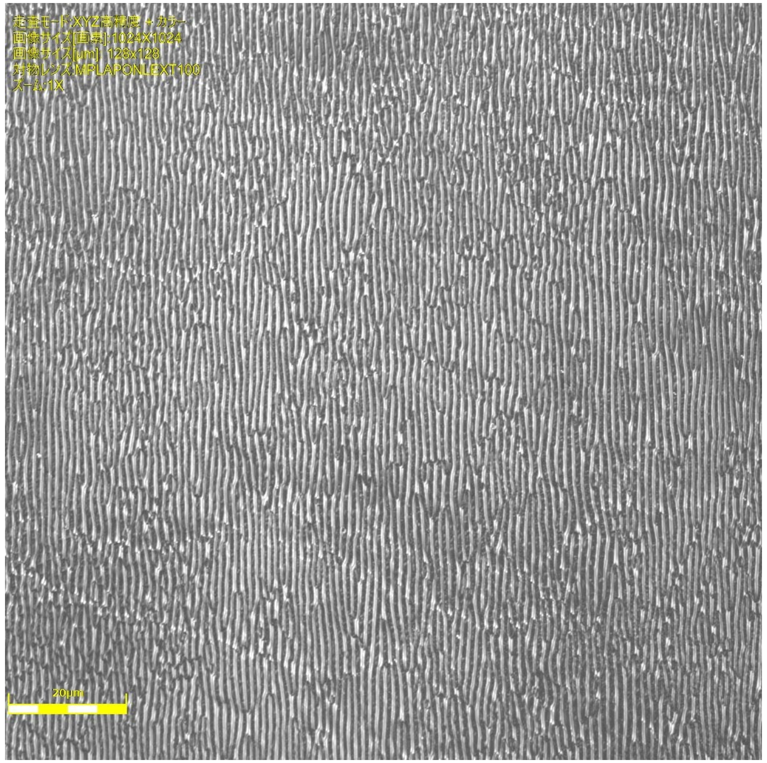

MIR-FEL

Original figures of Fig. 3 (TEM images of the LIPSS induced by Ti: sapphire laser)

a

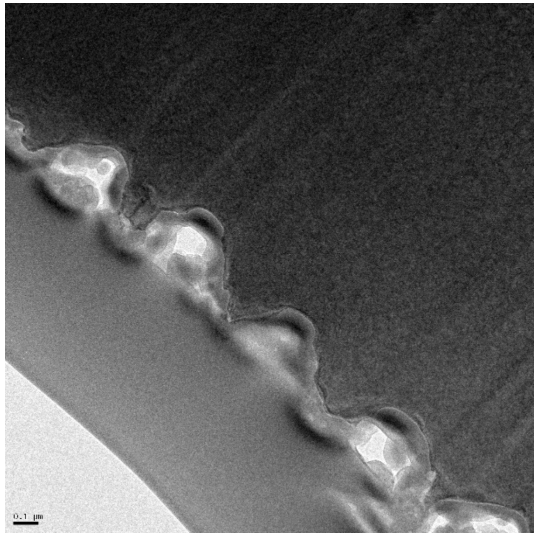

b

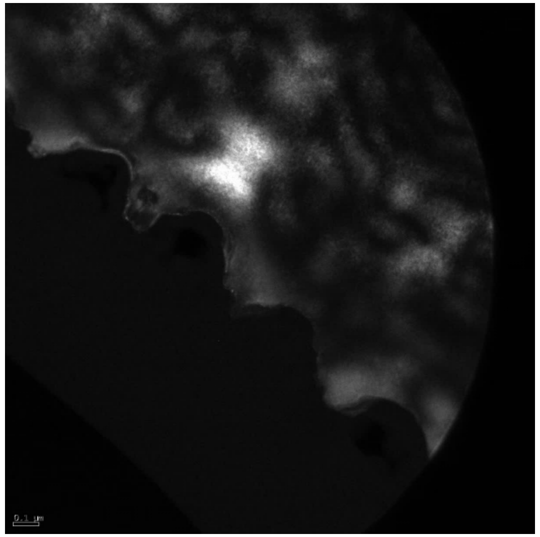

c

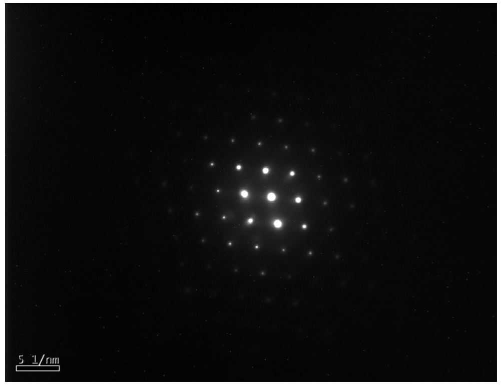

d

5nm^{-1}

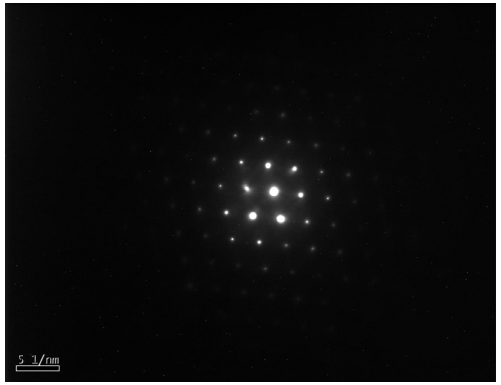

e

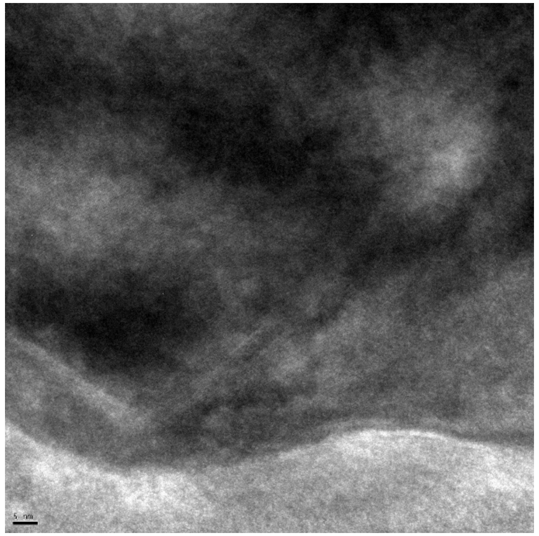

Original figures of Fig. 4 (TEM images of the LIPSS induced by MIE-FEL)

a

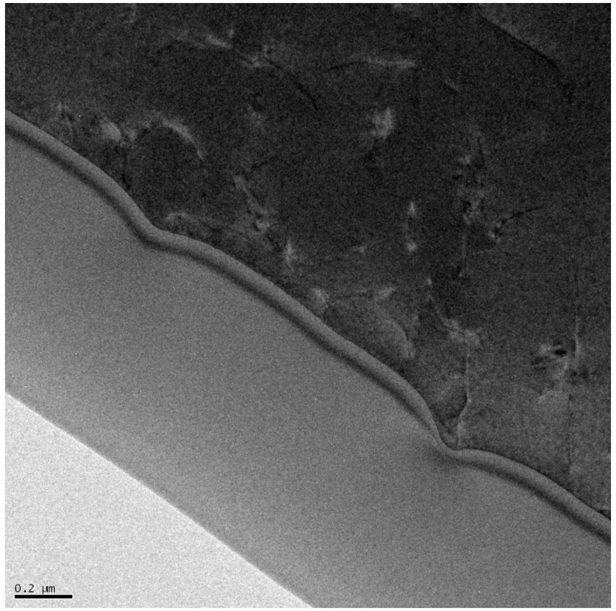

b

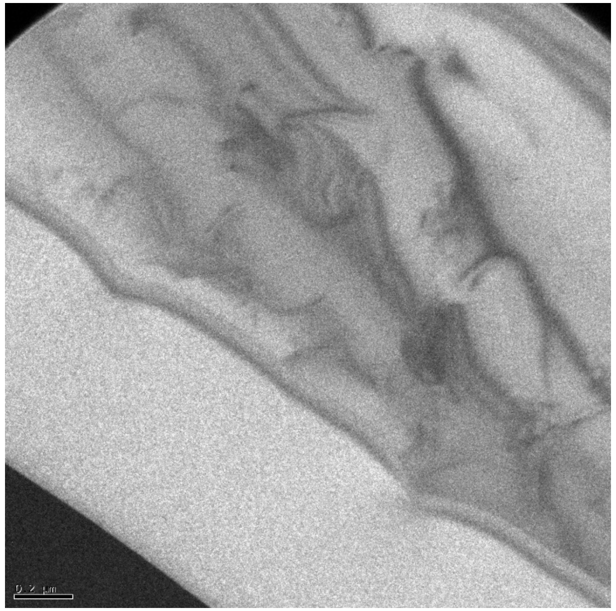

c

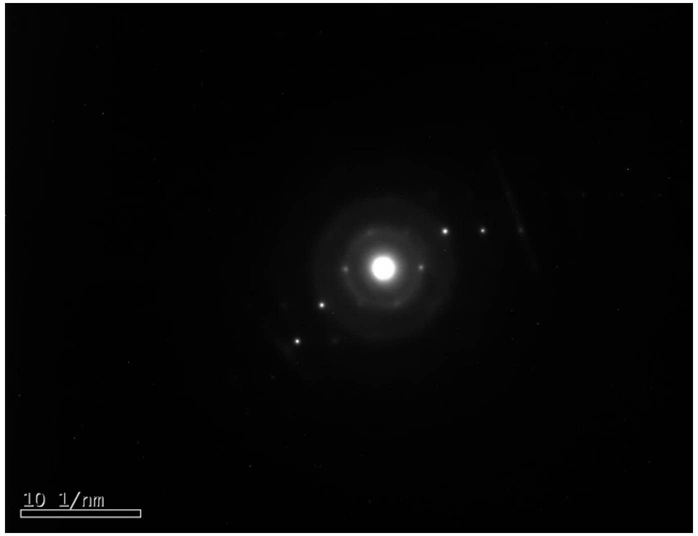

d

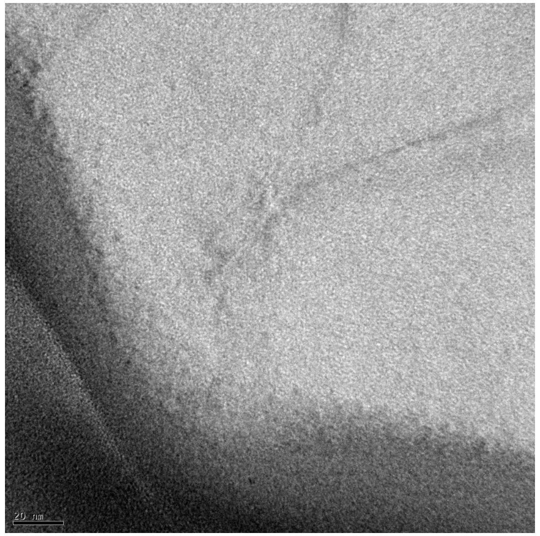

Original figure of Fig. 5 (two-dimensional XRD pattern)

a

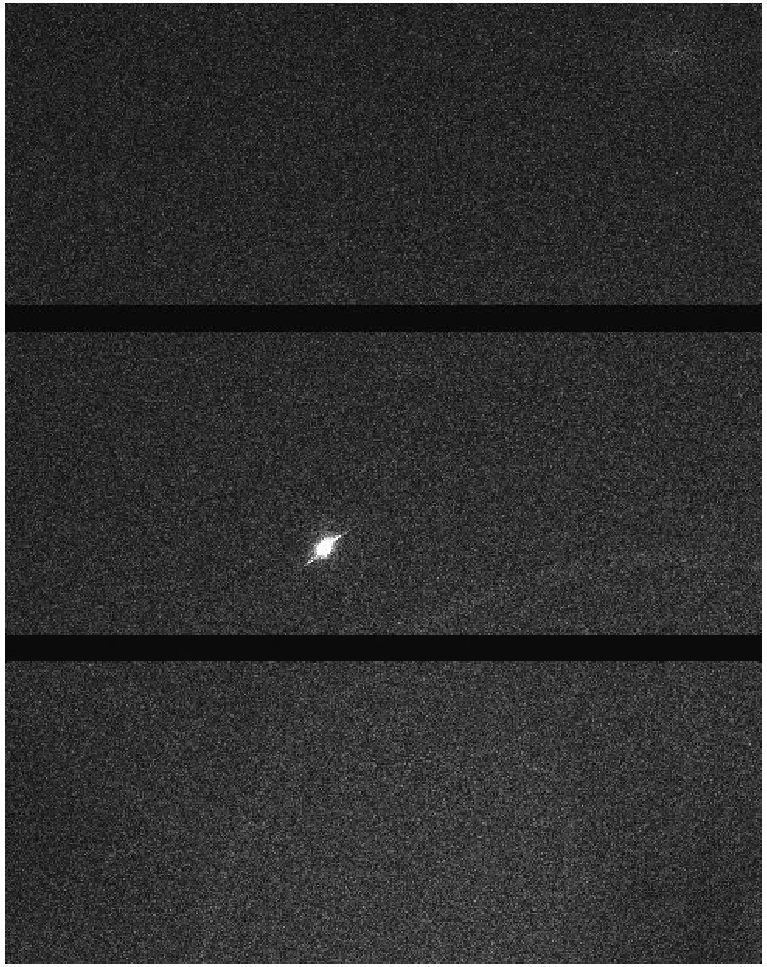

Data of Fig. 5 (the lattice spacing-intensity profiles of the LIPSS formed by Ti: sapphire laser and MIR-FEL)

b

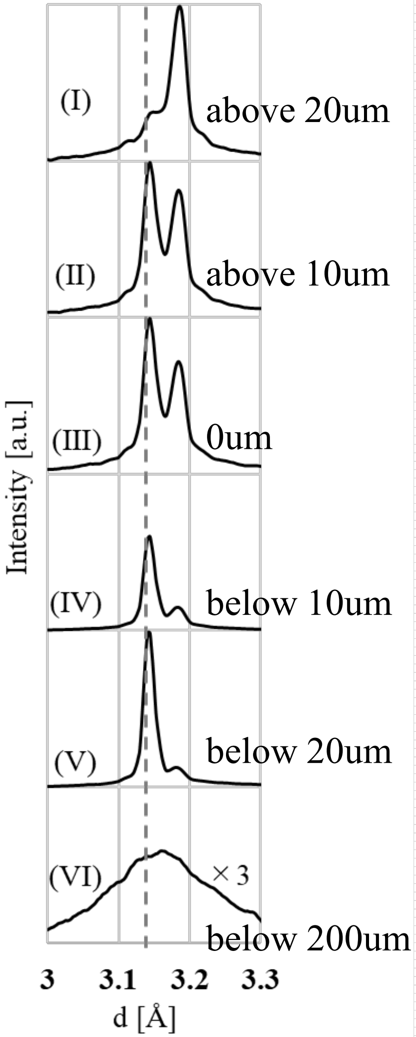

Ti: sapphire laser

(I)above 20um

2θ[°]	d[Å]	count[a.u.	normalize	for graph
7.15	3.311501	4.93233	0.050192	5.050192
7.155	3.30919	5.030208	0.051188	5.051188
7.16	3.306882	5.205539	0.052972	5.052972
7.165	3.304577	5.431915	0.055275	5.055275
7.17	3.302276	5.637357	0.057366	5.057366
7.175	3.299978	5.615803	0.057147	5.057147
7.18	3.297682	5.64697	0.057464	5.057464
7.185	3.295391	5.896309	0.060001	5.060001
7.19	3.293102	6.230377	0.063401	5.063401
7.195	3.290817	6.30017	0.064111	5.064111
7.2	3.288534	6.343164	0.064548	5.064548
7.205	3.286255	6.434877	0.065482	5.065482
7.21	3.283979	6.577325	0.066931	5.066931
7.215	3.281706	6.727484	0.068459	5.068459
7.22	3.279437	6.792659	0.069122	5.069122
7.225	3.27717	6.79483	0.069145	5.069145
7.23	3.274907	6.896681	0.070181	5.070181
[illegible]	[illegible]	[illegible]	[illegible]	[illegible]

(II)above 10um

2θ[°]	d[Å]	count[a.u.	normalize	for graph
7.15	3.311501	8.90578	0.063861	4.063861
7.155	3.30919	9.048376	0.064884	4.064884
7.16	3.306882	9.239789	0.066257	4.066257
7.165	3.304577	9.408422	0.067466	4.067466
7.17	3.302276	9.367066	0.067169	4.067169
7.175	3.299978	9.374627	0.067224	4.067224
7.18	3.297682	9.496646	0.068098	4.068098
7.185	3.295391	9.532364	0.068355	4.068355
7.19	3.293102	9.542814	0.06843	4.06843
7.195	3.290817	9.664817	0.069304	4.069304
7.2	3.288534	9.721161	0.069708	4.069708
7.205	3.286255	9.767434	0.07004	4.07004
7.21	3.283979	9.951982	0.071364	4.071364
7.215	3.281706	10.10601	0.072468	4.072468
7.22	3.279437	10.10164	0.072437	4.072437
7.225	3.27717	10.34214	0.074161	4.074161
7.23	3.274907	10.69579	0.076697	4.076697
[illegible]	[illegible]	[illegible]	[illegible]	[illegible]

(III)0um

2θ[°]	d[Å]	count[a.u	normalize	for graph
7.15	3.311501	8.510978	0.053811	3.053811
7.155	3.30919	8.586363	0.054288	3.054288
7.16	3.306882	8.792491	0.055591	3.055591
7.165	3.304577	9.037861	0.057143	3.057143
7.17	3.302276	9.189835	0.058103	3.058103
7.175	3.299978	9.479807	0.059937	3.059937
7.18	3.297682	9.785887	0.061872	3.061872
7.185	3.295391	9.98005	0.0631	3.0631
7.19	3.293102	9.907481	0.062641	3.062641
7.195	3.290817	9.86819	0.062392	3.062392
[illegible]	[illegible]	[illegible]	[illegible]	[illegible]

(IV)below 10um

2θ[°]	d[Å]	count[a.u	normalize	for graph
7.15	3.311501	7.663753	0.011277	2.011277
7.155	3.30919	7.871067	0.011582	2.011582
7.16	3.306882	8.011762	0.011789	2.011789
7.165	3.304577	8.275191	0.012177	2.012177
7.17	3.302276	8.643778	0.012719	2.012719
7.175	3.299978	8.834457	0.013	2.013
7.18	3.297682	9.017958	0.01327	2.01327
7.185	3.295391	9.258381	0.013624	2.013624
7.19	3.293102	9.555669	0.014061	2.014061
7.195	3.290817	9.726059	0.014312	2.014312
[illegible]	[illegible]	[illegible]	[illegible]	[illegible]

(V)below 20um

2θ[°]	d[Å]	count[a.u	normalize	for graph
7.15	3.311501	13.91452	0.020332	1.020332
7.155	3.30919	14.09176	0.020591	1.020591
7.16	3.306882	14.13145	0.020649	1.020649
7.165	3.304577	14.21388	0.020769	1.020769
7.17	3.302276	14.67718	0.021446	1.021446
7.175	3.299978	15.19825	0.022207	1.022207
7.18	3.297682	15.6138	0.022815	1.022815
7.185	3.295391	16.00425	0.023385	1.023385
7.19	3.293102	16.49247	0.024099	1.024099
7.195	3.290817	16.91225	0.024712	1.024712
[illegible]	[illegible]	[illegible]	[illegible]	[illegible]

(VI)below 200um

2θ[°]	d[Å]	count[a.u	normalize	for graph
7.15	3.311501	4.649181	0.044629	[illegible]
7.155	3.30919	4.619431	0.044343	[illegible]
7.16	3.306882	4.773152	0.045819	[illegible]
7.165	3.304577	5.231336	0.050217	[illegible]
7.17	3.302276	5.659139	0.054324	[illegible]
7.175	3.299978	6.085433	0.058416	[illegible]
7.18	3.297682	6.461564	0.062026	[illegible]
7.185	3.295391	6.910945	0.06634	[illegible]
7.19	3.293102	7.390523	0.070944	[illegible]
7.195	3.290817	7.658659	0.073517	[illegible]
[illegible]	[illegible]	[illegible]	[illegible]	[illegible]

c

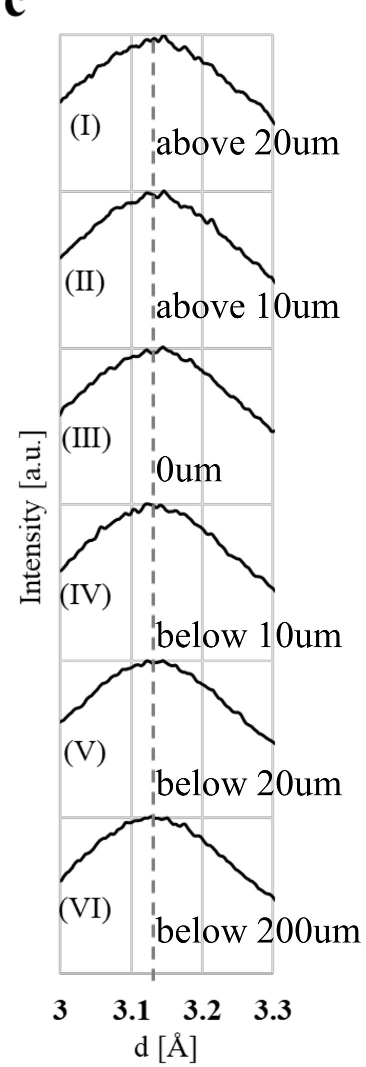

MIR-FEL

(I)above 20um

7.155	3.30919	96.7002	0.433666	6.433666
7.16	3.306882	95.67367	0.429062	6.429062
7.165	3.304577	94.63818	0.424419	6.424419
7.17	3.302276	95.66963	0.429044	6.429044
7.175	3.299978	98.63191	0.442329	6.442329
7.18	3.297682	102.7917	0.460984	6.460984
7.185	3.295391	106.1076	0.475855	6.475855
7.19	3.293102	109.7542	0.492209	6.492209
7.195	3.290817	113.37	0.508424	6.508424
7.2	3.288534	114.2941	0.512568	6.512568
7.205	3.286255	118.3631	0.530816	6.530816
[illegible]	[illegible]	[illegible]	[illegible]	[illegible]

(II)above 10um

7.155	3.30919	114.0399	0.408424	5.408424
7.16	3.306882	112.9185	0.404408	5.404408
7.165	3.304577	113.5936	0.406826	5.406826
7.17	3.302276	116.8949	0.41865	5.41865
7.175	3.299978	121.2243	0.434155	5.434155
7.18	3.297682	125.8046	0.450559	5.450559
7.185	3.295391	125.9761	0.451173	5.451173
7.19	3.293102	127.6641	0.457219	5.457219
7.195	3.290817	130.7459	0.468256	5.468256
7.2	3.288534	133.0049	0.476346	5.476346
7.205	3.286255	137.811	0.493559	5.493559
[illegible]	[illegible]	[illegible]	[illegible]	[illegible]

(III)0um

7.155	3.30919	161.5128	0.450619	4.450619
7.16	3.306882	165.7711	0.462499	4.462499
7.165	3.304577	166.7062	0.465108	4.465108
7.17	3.302276	166.465	0.464436	4.464436
7.175	3.299978	164.7757	0.459722	4.459722
7.18	3.297682	165.6204	0.462079	4.462079
7.185	3.295391	171.0679	0.477278	4.477278
7.19	3.293102	175.9055	0.490774	4.490774
7.195	3.290817	179.1958	0.499954	4.499954
7.2	3.288534	182.4237	0.50896	4.50896
7.205	3.286255	185.7575	0.518261	4.518261
[illegible]	[illegible]	[illegible]	[illegible]	[illegible]

(IV)below 10um

7.155	3.30919	169.1003	0.425259	3.425259
7.16	3.306882	171.027	0.430104	3.430104
7.165	3.304577	173.2051	0.435582	3.435582
7.17	3.302276	177.9418	0.447494	3.447494
7.175	3.299978	182.5137	0.458991	3.458991
7.18	3.297682	185.8386	0.467353	3.467353
7.185	3.295391	188.6193	0.474346	3.474346
7.19	3.293102	192.0868	0.483066	3.483066
7.195	3.290817	197.1364	0.495765	3.495765
7.2	3.288534	202.2865	0.508717	3.508717
7.205	3.286255	207.7424	0.522437	3.522437
[illegible]	[illegible]	[illegible]	[illegible]	[illegible]

(V)below 20um

7.155	3.30919	200.1392	0.450685	2.450685
7.16	3.306882	204.3082	0.460073	2.460073
7.165	3.304577	206.9984	0.466131	2.466131
7.17	3.302276	209.018	0.470679	2.470679
7.175	3.299978	213.1394	0.47996	2.47996
7.18	3.297682	216.1157	0.486662	2.486662
7.185	3.295391	218.7328	0.492555	2.492555
7.19	3.293102	221.5058	0.4988	2.4988
7.195	3.290817	224.4113	0.505342	2.505342
7.2	3.288534	228.0565	0.513551	2.513551
7.205	3.286255	231.7575	0.521885	2.521885
[illegible]	[illegible]	[illegible]	[illegible]	[illegible]

(VI)below 200um

3.30919	0.449741	1.449741	[illegible]
3.306882	0.453177	1.453177	[illegible]
3.304577	0.458274	1.458274	[illegible]
3.302276	0.469301	1.469301	[illegible]
3.299978	0.485361	1.485361	[illegible]
3.297682	0.496068	1.496068	[illegible]
3.295391	0.503343	1.503343	[illegible]
3.293102	0.506352	1.506352	[illegible]
3.290817	0.508128	1.508128	[illegible]
3.288534	0.512453	1.512453	[illegible]
3.286255	0.522032	1.522032	[illegible]
[illegible]	[illegible]	[illegible]	[illegible]
